# Increased Anxiety and Depression Symptoms in Post-Acute Care Patients with Stroke during the COVID-19 Pandemic

**DOI:** 10.3390/ijerph19010162

**Published:** 2021-12-24

**Authors:** Hsiang-Yun Chou, Yu-Chun Lo, Ya-Wen Tsai, Chia-Li Shih, Chieh-Ting Yeh

**Affiliations:** 1 Department of Rehabilitation, An Nan Hospital, China Medical University, Tainan 709204, Taiwan; chiali771223@gmail.com; 2 The Ph.D. Program for Neural Regenerative Medicine, College of Medical Science and Technology, Taipei Medical University, Taipei 11031, Taiwan; aricalo@tmu.edu.tw; 3 Department of Nursing, An Nan Hospital, China Medical University, Tainan 709204, Taiwan; ritayeh0823@gmail.com

**Keywords:** post-acute care, stroke, rehabilitation, functional outcome, mental health, anxiety, depression, COVID-19

## Abstract

This study aimed to explore the quality and stability of post-acute care for patients with stroke, including their functional outcomes, mental health and medical care in Taiwan during the COVID-19 pandemic. In this retrospective case–control study—based on propensity score matching—we assessed 11 patients admitted during the pandemic period (in 2021) and 11 patients admitted during the non-pandemic period (in 2020). Functional outcomes, including the scores of the modified Rankin Scale, Barthel Index, EuroQoL-5 Dimension, Lawton–Brody instrumental activities of daily living, Berg Balance Scale, 5-metre walking speed and 6-min walking distance, were determined. Data on the length of acute care, length of post-acute care, destination after discharge and 14-days readmission were used to evaluate the quality of medical care. The Wilcoxon signed-rank test was used to compare functional performance before and after rehabilitation. The pandemic group showed no significant improvement in the scores of EuroQoL-5 Dimension, a self-reported health status assessment (*p* = 0.13), with the anxiety or depression dimension showing a negative effect (r = 0.21). Post-acute care programmes can efficiently improve the functional performance of patients with stroke during the COVID-19 pandemic in Taiwan. Mental health should therefore be simultaneously maintained while rehabilitating physical function.

## 1. Introduction

Since 2019, the world has witnessed people struggle against COVID-19 daily. Taiwan, which has a population of 23.5 million, was able to successfully resist the COVID-19 outbreak throughout 2020 [1]. Unexpectedly, a COVID-19 outbreak occurred in Taiwan in May 2021. Based on the Taiwan Center for Disease Control’s ‘Pandemic Alert Standards and Response Measures’, the government announced that Taiwan would be placed on third-level alert status starting 19 May 2021. The epidemic alert status is divided into levels 1, 2, 3 and 4, with higher levels corresponding to stricter control measures. From May 19, the government mandated the wearing of facial masks in public, cessation of gatherings involving more than 5 people indoors and 10 people outdoors and maintaining physical distance. To prevent excessive load on medical resources, non-essential medical activities, such as general health examinations, medical cosmetics and rehabilitation therapy, were discouraged. However, certain individuals, such as patients with stroke, still required urgent medical intervention and hospitalisation.

Stroke survivors usually experience severe functional loss, which reduces physical activity and social participation and increases socioeconomic burden [2,3]. In a recent review study, a decrease in emergency care for ischemic stroke was observed during the COVID-19 outbreak, leading to an increase in disability and deaths, which will impose additional economic burdens on the healthcare system and society [4]. Timely inpatient rehabilitation can effectively reduce the mortality and disability rates and decrease subsequent socioeconomic burdens [5,6,7]. Therefore, physicians should actively engage in rehabilitation interventions following the stabilisation of the patient’s medical condition after the acute phase. In 2014, the Taiwan National Health Insurance Administration launched its post-acute care cerebrovascular disease (PAC-CVD) programme [8], which emphasises high-intensity inpatient rehabilitation facilities (IRFs, 3–5 times daily) and a medical referral system for long-term care after discharge from community hospitals [8]. The PAC programme utilises per diem reimbursement, which differs from fee-for-service, to reduce daily expenditure [8]. A study showed that the length of stay (LOS) of PAC-CVD patients in Taiwan is slightly more than 1 month [9]. Despite the lack of recent data indicating whether patients with stroke have high COVID-19 infection rates, factors such as old age, obesity, smoking, hypertension, cardiovascular disease and diabetes have been strongly associated with increased infection rates in these patients [10,11,12,13]. Research has suggested that low levels of physical activity are associated with the risk of COVID-19, severe illness from COVID-19 and COVID-19-related death [14]. Therefore, motor function recovery may prevent COVID-19 and death.

Besides physical function, mental health requires attention. A recent study showed that people experienced substantial psychological impact in the form of stress, anxiety and depression during the COVID-19 outbreak [15]. The PAC programme significantly improves the motor function status, activities of daily living, quality of life, oral function and mental health of patients with stroke [8,16,17]. Since 2014, the PAC programme has never experienced an infectious disease outbreak. This study therefore aimed to investigate the quality and stability of the PAC-CVD programme in terms of improving functional outcomes and mental health status during the COVID-19 pandemic.

## 2. Materials and Methods

### 2.1. Study Design and Patients

The study population comprised all patients admitted with stroke to the PAC ward of a regional hospital that has expertly provided PAC service for >5 years. The patients were categorised into two groups: a non-pandemic group (admitted in 2020) and a pandemic group (admitted between mid-May to early August of 2021). The inclusion criteria of the PAC-CVD programme were as follows [16]: (1) admitted within 30 days after acute onset of cerebrovascular disease; (2) no deterioration in blood pressure, heart rate, body temperature and neurological condition for more than 72 h; (3) moderate-to-moderately-severe motor dysfunction, modified Rankin Scale (mRS) scores of 3–4 [18] and (4) basic cognition, learning ability and willingness. The covariates included patient demographics (age and sex), clinical attributes (stroke type, hypertension, coronary artery disease, diabetes mellitus, hyperlipidaemia and previous stroke), common risk factors (smoking and drinking) and pre-rehabilitation functional status. Ultimately, 11 patients in the pandemic group were compared with 11 patients in the non-pandemic group. All PAC patients completed the pre-rehabilitation and discharge-rehabilitation assessments. The study protocol was approved by the institutional review board of Taiwan Municipal An Nan Hospital-China Medical University (TMANH110-REC032). Before enrolment, all participants provided written informed consent.

### 2.2. Functional Status Instruments and Chart Review

The mRS, Barthel Index (BI), EuroQoL-5 Dimension (EQ-5D), Lawton–Brody instrumental activities of daily living (IADL), Berg Balance Scale (BBS), 5 m walking speed (5MWS) and 6-min walking distance (6MWD) were used to assess functional performance. mRS scores of 0, 1, 2, 3, 4, 5 and 6 correspond to no symptoms, no significant disability, slight disability, moderate disability, moderately severe disability, severe disability and death, respectively [19]. The BI scores indicate functional disability for activities of daily living, ranging from 0 (completely dependent) to 100 (independent) [20]. The EQ-5D, a self-reported health status assessment, comprises five dimensions corresponding to mobility, self-care, routine activities, pain or discomfort and anxiety or depression as part of a total health state, with higher scores indicating more severe or frequent problems [21]. The IADL, which comprises eight items corresponding to making phone calls, shopping, preparing food, housekeeping, laundering, taking pills, using transportation and managing finances, is used to evaluate how patients engage in activities of daily living [22]. The BBS, which comprises 14 items, is used to evaluate functional balance, with each item rated from 0 (poor balance) to 4 (good balance) [23]. The 5MWS assesses longitudinal changes in walking speed after stroke [24]. The 6MWD evaluates walking ability, with studies suggesting that accomplishing a distance of ≥205 m better defines community ambulation ability compared with walking speed [25].

The following patient data were obtained from medical records: age, sex, type of stroke, comorbidities (hypertension, coronary artery disease, diabetes mellitus and hyperlipidaemia), previous stroke, common risk factors (smoking and drinking), length of acute care, length of post-acute care, total score for each functional status measure before and after rehabilitation, readmission after 14 days and destination after discharge.

### 2.3. Public Health Policies for Rehabilitation

The patient and caregiver needed to have a negative polymerase chain reaction (PCR) test 3 days before being transferred to the regional hospital. Isolation equipment was worn, and an ambulance was used for transfers between hospitals to reduce the risk of exposure to infection. During hospitalisation, the patients were admitted to a ward dedicated to patients with stroke, accompanied by the same caregiver, and were instructed to avoid leaving the hospital arbitrarily. Family visits were limited to online video calls. Both the patients and caregivers were required to monitor their body temperature daily. Rehabilitation interventions were conducted in a separate room, with the equipment thoroughly disinfected after use. The stroke patient engaged in a highly intensive PAC programme, defined as a high frequency of rehabilitation (3–5 times daily), including physical therapy, occupational therapy, and speech therapy [8,26]. This significantly increased the period of contact with different healthcare workers. Healthcare workers were at high risk of infection during the COVID-19 pandemic, following WHO’s recommendations for the frequent washing of soiled hands or the use of sanitizer for unsoiled hands to help reduce the spread of diseases [27]. The medical staff, including doctors, nurses, physical therapists, occupational therapists and speech therapists, were required to receive at least the first dose of the COVID-19 vaccine, report contact and cluster history and record their body temperature daily. Population-based measures, including the wearing of face masks, personal hygiene and appropriate physical distancing, were strictly implemented during rehabilitation [28].

### 2.4. Statistical Analysis

Statistical analysis was conducted using SPSS version 26.0 (IBM Corp., Armonk, NY, USA). Baseline clinical and descriptive statistics were utilised to depict the patients’ demographics. To prevent non-comparability between the groups due to distortions in the estimation of the treatment effect, propensity score matching (PSM) was estimated using a multivariable logistic regression model adjusted for observed covariates, assigning one matched control for each patient admitted in 2020 according to the patient-level minimised systematic differences in baseline characteristics when comparing both groups [29,30,31]. The calliper matching method was used for PSM between the non-pandemic group and the pandemic group, and the match tolerance was set “0.2” for 1:1 matching. The independent samples t-test and Mann–Whitney U test were performed to determine whether significant differences existed between the groups, with data presented as means and standard deviations, proportions or medians. The Wilcoxon signed-rank test was used to compare functional performance before and after rehabilitation, with data presented as medians and interquartile ranges. Effect sizes (ES) were estimated to determine the magnitude of change in the functional outcome scores. A clinically meaningful effect was defined as an absolute value ES of ≥0.1, with an ES of ≥0.1, ≥0.3 and ≥0.5 indicating a small, moderate and large effect, respectively [32]. All tests were two-sided, and a *p*-value of <0.05 indicated statistical significance.

## 3. Results

Eighty-eight patients were admitted to the PAC programme at a regional hospital during the non-pandemic period of 2020, among whom three dropped out due to working medical conditions and another three requested withdrawal due to personal reasons. Therefore, 82 patients completed the programme and all evaluations during the non-pandemic period. During the pandemic period in 2021, when Taiwan entered the third-level alert status, 12 patients were admitted to the PAC programme. One patient dropped out due to the family’s request. Ultimately, 11 patients completed the programme and all evaluations during the pandemic period. Before performing PSM, the non-pandemic group (*n* = 82) was significantly older than the pandemic group (*n* = 11) (*p* < 0.05). No significant differences in the male:female ratio, clinical attributes, common risk factors and pre-rehabilitation functional status were observed between the groups. After performing PSM, the non-pandemic (*n* = 11) and pandemic groups (*n* = 11) had no significant difference in age, sex, clinical attributes, common risk factors and pre-rehabilitation functional status scores (Table 1).

After PAC intervention, the non-pandemic group showed significant improvements in all functional status measures (admission versus before discharge), including mRS, BI, EQ-5D, IADL, BBS, 5MWS and 6MWD. The pandemic group showed significant improvements in most measures of functional status after PAC intervention, including mRS, BI, IADL, BBS, 5MWS and 6MWD. EQ-5D scores were not significantly different in the pandemic group (*p* = 0.13) (Table 2).

The magnitude of change in the functional outcome scores was expressed in ES. In the non-pandemic group, BBS and BI had the largest training effect (ES: 0.63, large effect), whereas mRS (ES: 0.53, large effect) had the smallest training effect. In the pandemic group, BBS (ES: 0.63, large effect) had the largest training effect, whereas EQ-5D (ES: 0.32, moderate effect) had the smallest training effect. The largest difference between both groups was in EQ-5D (ES1-ES2: 0.23; ES1 and ES2 indicate the change in functional outcomes in the pandemic and non-pandemic groups, respectively), whereas the smallest difference between the groups was in BBS (ES1-ES2: 0). No significant difference in mRS, BI, IADL, BBS, 5MWS and 6WMD was observed between the groups (Table 3).

The five dimensions (mobility, self-care, usual activities, pain or discomfort and anxiety or depression) of the EQ-5D manifest the patients’ self-reported mental health status. The pandemic group showed no significant difference in the five dimensions of patient self-expression after PAC intervention, with the self-care dimension having the largest absolute ES value (ES:0.4, moderate effect) and mobility, pain or discomfort and anxiety or depression having the smallest absolute ES value (ES:0.21, small effect; anxiety or depression showed a negative real number). In the non-pandemic group, significant differences in three dimensions (mobility, *p* < 0.01; self-care, *p* = 0.04; usual activities, *p* < 0.01) were observed after PAC intervention, with mobility and usual activities having the largest absolute ES value (ES: 0.56, large effect) and pain or discomfort having the smallest absolute ES value (ES: 0.21, small effect). The largest difference between the groups was in the anxiety or depression dimension (ES1-ES2: 0.51), whereas the smallest difference was in the pain or discomfort dimension (ES1-ES2: 0) (Table 4).

The quality of medical care, which was assessed using acute care LOS, PAC LOS, destination after discharge, readmission after 14 days and 6MWD ≥ 205 m, did not significantly differ between the groups (Table 5).

## 4. Discussion

This study explored the quality and stability of post-acute care for patients with stroke, including their functional outcomes, mental health and medical care, in Taiwan during the COVID-19 pandemic. The present study was the first to assess the mental health of PAC patients using the self-reported EQ-5D during the COVID-19 pandemic. The pandemic group showed no significant improvement in EQ-5D scores, and EQ-5D scores had the largest difference between the pandemic and non-pandemic groups. The PAC-CVD programme serves as a transitional phase between discharge from medical institutions and returning home or enrolment in long-term care systems [33]. This programme aims to reduce the disability and medical costs of patients with stroke and promote the rehabilitation of their functional ability. Most previous studies focused on the functional recovery of patients with stroke while ignoring their mental health [26,34]. Further analysis of the five dimensions of EQ-5D showed that anxiety or depression tended to be more severe during the pandemic. Estimates have shown that approximately 20% to 25% of patients develop anxiety disorders after stroke [35], which affects their quality of life [36]. A depressed or dysphoric mood affects approximately 30% of stroke survivors [37], reducing the motivation for rehabilitation and promoting poorer functional outcomes or even increased risk for suicide [38,39]. In a recent study, although depression did not significantly worsen, higher rates of anxiety were observed in patients with stroke during the COVID-19 pandemic [40]. After reviewing our patients’ medical records, we found that the 11 patients were not diagnosed with post-stroke depression during the pandemic. However, those who experienced stroke during the COVID-19 pandemic may have exhibited some degree of anxiety or depressive symptoms, especially the younger patients. The mean age of the patients included in our study was 53 years, which was significantly younger than that in previous studies [9,26]. This difference may be attributed to the following reasons: as the risk for more severe COVID-19-associated outcomes increases with age, most patients aged ≥65 years chose to return home or preferred home-based rehabilitation instead of inpatient rehabilitation [13]. Telerehabilitation could potentially be taught and practiced at home during the COVID-19 pandemic via cell phones and Skype, or even be combined with modern technology, including virtual reality (VR) and robotic assistance [41,42]. Considering that rehabilitation is less effective in those aged ≥65 years than in those aged <65 years, patients and their families had a relatively negative perception of rehabilitation during the pandemic and showed reduced willingness to undergo the PAC programme [17]. Young and middle-aged patients were considered to have a longer lifespan, to be more productive, to be the source of household income and to have greater motivation for participating in rehabilitation [17]; the medical staff perceived younger patients with stroke who engaged in high-intensity rehabilitation to have good recovery benefits and actively persuaded them to undergo the PAC programme. COVID-19 has led to serious losses in many economies around the world, especially among low-income populations, causing a sharp rise in unemployment and poverty rates [43]. Besides physical anxiety, younger patients who were the source of household income perceived the COVID-19-related panic [15] and the lower household income caused by physical disabilities or the pandemic as stressors [44]. On the contrary, the different level of financial assets was associated with disability in stroke patients. Lower household income reflected a higher level of disability and increased the gap in participation and activities [45]. In addition to the huge financial burden of the long-term care of stroke, the burden of caregivers was intensive, especially the family caregiver during the COVID-19 pandemic [46]. The Taiwan government adopted strict social distancing measures and prevented excessive load on medical resources during the COVID-19 pandemic, resulting in the suspension of certain medical services such as rehabilitation centres. In addition to the provision of essential daily care, the family caregiver also assumed the additional role of a rehabilitation therapist at home, which caused not only physical but psychological burdens [46]. 

The PAC programme mainly emphasises physical function recovery and psychological intervention. Although it can improve mental health, psychological problems were only revealed during the outbreak. During the pandemic, the public experienced anxiety, stress, fear, uncertainty and insecurity [47], indicating the need for more psychological support among patients with stroke, who are considered a vulnerable population. Thus, the PAC phase helps in the early recognition of psychological problems and the provision of timely interventions. This may be attributed to the clinical staff’s adjustment of the patient-personalised plan, assessment of mental health status and treatment of patients during this transitional phase, which would prepare them for their return to society. The clinical staff may even extend their service until after discharge from the hospital, following up patients during outpatient rehabilitation or through telephone.

This study revealed that during the outbreak, PAC-CVD patients showed no significant difference in functional recovery improvement compared with those included in previous studies under common conditions. No significant improvement was observed in the self-reported dimensions of mobility, self-care and usual activities during the outbreak. Evidence has shown that depressive syndrome is associated with lower general health perception and that physical functional disability affects the quality of life [48]. The lower general health perception may impede improvements in medical condition and the recovery of motor function. Thus, this programme is robust and effective in rehabilitating functional abilities among post-stroke patients during infectious disease outbreaks; psychological problems promote a vicious circle between physical and mental health. This suggests the importance of monitoring mental health status and preventing negative psychological consequences.

Case managers in institutions should be involved in the referral process. During this COVID-19 wave, they play an important role, including preparation before transfer, confirming negative PCR tests 3 days before admission and contacting the institution to which the patient is to be transferred before discharge. We found no significant difference in acute LOS between both groups. Besides medical treatment for acute stroke at the medical centre, including the preparation of the PAC programme for referral to the regional hospital, the acute LOS could indicate the timeliness and continuity of the PAC programme during the pandemic. The similar PAC LOS in both groups under the per diem reimbursement system suggests no increase in economy with efficient functional recovery during the pandemic. None of the patients were hospitalised within 14 days, and most returned home after discharge from the regional hospital. Previous studies determined the destination of the patients after discharge, which is important for our research considering the young age of our cohort. Nearly 30% of these young patients demonstrated a 6MWD ≥ 205 m after high-intensity rehabilitative interventions, which could indicate their ability for community ambulation [25].

This article was drafted during the period wherein Taiwan had been downgraded to second-level alert, which may have decreased the level of mental stress among the patients with stroke. Future research should focus on changes in psychological conditions to provide more substantial guidance for medical institutions. Fighting a pandemic is akin to running a marathon—one must never lower their guard. The end of the pandemic cannot be predicted. However, all individuals should adjust to the changes, even patients with stroke.

This study has some limitations. First, the sample size was small and was obtained from a single centre, which was one of the institutions in southern Taiwan with the greatest number of PAC cases. Notably, the hospitalisation period coincided with the small-scale cluster infection of COVID-19 in this district. Adopting a strict medical isolation public health policy in the case of limited medical capacity and maintaining the quality of the PAC-CVD programme are challenging. This study was expected to raise the awareness of the physical and psychological health of stroke patients during the COVID-19 pandemic, although the small sample size may not represent a macro-level analysis. Second, this was a retrospective study that reviewed medical records, observed phenomena and made clinical recommendations. Third, only PAC-CVD cases were enrolled due to the medical load reduction policy.

## 5. Conclusions

This study highlighted the quality and stability of the PAC-CVD programme for patients with stroke in medical care and improving functional outcomes. The PAC-CVD programme focuses on the recovery of the motor function of stroke patients, but COVID-19 has increased people’s health awareness and psychological pressure. While restoring the motor function of the patients, attention should also be paid to their mental health and the provision of psychological support. This study also raised awareness of the huge burden on the economy and rehabilitation care for stroke patients during the COVID-19 pandemic. In addition to verifying the efficiency and stability of the PAC-CVD programme during the pandemic, this study could serve as a reference for framing public health policies for rehabilitation institutions.

## Figures and Tables

**Table 1:** Patients’ characteristics before and after propensity score matching (PSM).

		Before Propensity Score Matching						After Propensity Score Matching					
Variables		Pandemic Group (*n* = 11)		Non-Pandemic Group (*n* = 82)		SMD	*p*	Pandemic Group (*n* = 11)		Non-Pandemic Group (*n* = 11)		SMD	*p*
Demographics		Mean (SD) or *n* (%)						Mean (SD) or *n* (%)					
Age, years		53.64 (15.85)		63.44 (14.65)		0.642	0.04 *	53.64 (15.85)		52.91 (17.71)		0.043	0.92
Sex	Female	2 (18.2%)		26 (31.7)		0.320	0.33	2 (18.2%)		2 (18.2%)		0	1.0
	Male	9 (81.8%)		56 (68.3%)				9 (81.8%)		9 (81.8%)			
Clinical Attributes													
Stroke Type						0.120	0.71					0	1.0
Ischemic		9 (81.8%)		63 (76.8%)				9 (81.8%)		9 (81.8%)			
Hemorrhagic		2 (18.2%)		19 (23.2%)				2 (18.2%)		2 (18.2%)			
Hypertension		10 (90.9%)		78 (95.1%)		0.152	0.57	10 (90.9%)		10 (90.9%)		0	1.0
Coronary Artery Disease		3 (27.3%)		20 (24.4%)		0.067	0.84	3 (27.3%)		3 (27.3%)		0	1.0
Diabetes Mellitus		2 (18.2%)		29 (35.4%)		0.382	0.22	2 (18.2%)		1 (9.1%)		0.252	0.56
Hyperlipidemia		6 (54.5%)		35 (42.7%)		0.235	0.46	6 (54.5%)		6 (54.5%)		0	1.0
Previous Stroke		3 (27.3%)		17 (21.5%)		0.137	0.63	3 (27.3%)		2 (18.2%)		0.206	0.63
Common Risk Factors													
Smoking		6 (54.5%)		16 (19.5%)		0.753	0.05	6 (54.5%)		6 (54.5%)		0	1.0
Drinking		3 (27.3%)		12 (14.6%)		0.289	0.29	3 (27.3%)		3 (27.3%)		0	1.0
Pre-rehabilitation Functional Status													
		Mean (SD)	Median (IQR)	Mean (SD)	Median (IQR)	SMD	*p*	Mean (SD)	Median (IQR)	Mean (SD)	Median (IQR)	SMD	*p*
mRS		3.55 (0.52)	4 (3–4)	3.65 (0.48)	4 (3–4)	0.20	0.52	3.55 (0.52)	4 (3–4)	3.64 (0.51)	4 (3–4)	0.175	0.67
BI		50.91 (15.12)	50 (40–65)	48 (20.58)	50 (35–61)	0.132	0.66	50.91 (15.12)	50 (40–65)	50 (18.17)	55 (40–60)	0.054	0.95
EQ-5D		8.82 (1.25)	8 (8–10)	8.95 (1.45)	8 (8–10)	0.096	0.77	8.82 (1.25)	8 (8–10)	8.64 (1.03)	8 (8–9)	0.157	0.85
IADL		1.09 (1.04)	1 (0–2)	1.24 (1.19)	1 (0–2)	0.134	0.69	1.09 (1.04)	1 (0–2)	1.36 (1.03)	2 (0–2)	0.261	0.51
BBS		22.73 (16.13)	20 (5–36)	18.04 (14.49)	11.5 (6–30.25)	0.306	0.32	22.73 (16.13)	20 (5–36)	22.45 (13.71)	23 (9–38)	0.019	0.95

SD: standard deviation; mRS: modified Rankin Scale; BI: Barthel Index; EQ-5D: EuroQoL-5 Dimension; IADL: Lawton–Brody instrumental activities of daily living; BBS: Berg Balance Scale; SMD: standardized mean difference. Values are expressed as mean (SD), median and interquartile range (IQR), or *n* %. * Statistically significant (*p* < 0.05).

**Table 2:** Total score for each functional status measure before and after the PAC program in both groups.

Measures	Pandemic Group (*n* = 11)			Non-Pandemic Group (*n* = 11)		
	Before PAC	After PAC	*p*	Before PAC	After PAC	*p*
mRS	4 (3–4)	3 (2–4)	0.02 *	4 (3–4)	2 (2–3)	0.01 *
BI	50 (40–65)	80 (60–100)	<0.01 *	55 (40–60)	85 (70–95)	<0.01 *
EQ-5D	8 (8–10)	8 (6–9)	0.13	8 (8–9)	7 (5–8)	<0.01 *
IADL	1 (0–2)	4 (0–5)	0.02 *	2 (0–2)	3 (2–4)	<0.01 *
BBS	20 (5–36)	52 (30–56)	<0.01 *	23 (9–38)	44 (27–50)	<0.01 *
5MWS	0 (0–0.21)	0.56 (0.13–1.22)	<0.01 *	0 (0–0)	0.42 (0–0.48)	0.01 *
6MWD	0 (0–100)	130 (40–416)	<0.01 *	0 (0–0)	150 (0–230)	0.01 *

PAC: post-acute care; mRS: modified Rankin Scale; BI: Barthel Index; EQ-5D: EuroQoL-5 Dimension; IADL: Lawton–Brody instrumental activities of daily living; BBS: Berg Balance Scale; 5MWS: 5-m walking speed; 6MWD: 6-min walking distance. Values are expressed as median and interquartile range (IQR), i.e., 4 (3–4) means the median is 4 and the interquartile range is from 3 to 4. * Statistically significant (*p* < 0.05).

**Table 3:** Differences in effect size (ES) in each functional status measure before and after the PAC program: comparison between different groups.

Measures	Pandemic Group (*n* = 11)			Non-Pandemic Group (*n* = 11)			
	Before PAC	After PAC	ES1	Before PAC	After PAC	ES2	ES1-ES
mRS	4 (3–4)	3 (2–4)	−0.50	4 (3–4)	2 (2–3)	−0.53	0.03
BI	50 (40–65)	80 (60–100)	0.57	55 (40–60)	85 (70–95)	0.63	−0.06
EQ-5D	8 (8–10)	8 (6–9)	−0.32	8 (8–9)	7 (5–8)	−0.55	0.23
IADL	1 (0–2)	4 (0–5)	0.52	2 (0–2)	3 (2–4)	0.61	−0.09
BBS	20 (5–36)	52 (30–56)	0.63	23 (9–38)	44 (27–50)	0.63	0
5MWS	0 (0–0.21)	0.56 (0.13–1.22)	0.57	0 (0–0)	0.42 (0–0.48)	0.54	0.03
6MWD	0 (0–100)	130 (40–416)	0.57	0 (0–0)	150 (0–230)	0.54	0.03

PAC: post-acute care; mRS: modified Rankin Scale; BI: Barthel Index; EQ-5D: EuroQoL-5 Dimension; IADL: Lawton–Brody instrumental activities of daily living; BBS: Berg Balance Scale; 5MWS: 5-m walking speed; 6MWD: 6-min walking distance; ES, effect size. Values are expressed as median and interquartile range (IQR), i.e., 4 (3–4) means the median is 4 and the interquartile range is from 3 to 4. ES1 and ES2 indicate the change in functional outcome in pandemic and non-pandemic groups, respectively. ES1-ES2 indicates the difference between both two groups.

**Table 4:** The five dimensions in EQ-5D before and after PAC program: comparison between different groups.

Dimensions	Pandemic Group (*n* = 11)				Non-Pandemic Group (*n* = 11)				
	Before PAC	After PAC	*p*	ES1	Before PAC	After PAC	*p*	ES2	ES1-ES
Mobility	2 (2–2)	2 (1–2)	0.32	−0.21	2 (2–2)	1 (1–2)	<0.01 *	−0.53	0.35
Self-Care	2 (2–2)	2 (1–2)	0.06	−0.40	2 (2–2)	2 (1–2)	0.04 *	−0.43	0.03
Usual Activities	2 (2–3)	2 (2–3)	0.08	−0.37	2 (2–3)	2 (1–2)	<0.01 *	−0.56	0.19
Pain or Discomfort	1 (1–1)	1 (1–1)	0.32	−0.21	1 (1–1)	1 (1–1)	0.32	−0.21	0
Anxiety or Depression	1 (1–1)	1 (1–1)	0.32	0.21	1 (1–1)	1 (1–1)	0.16	−0.30	0.51

PAC: post-acute care; EQ-5D: EuroQoL-5 Dimension; ES, effect size. ES1 means the change in functional outcome in the pandemic group; ES2 means the change in functional outcome in the non-pandemic group. ES1-ES2 indicates the difference between the two groups. Values are expressed as median and interquartile range (IQR), i.e., 4 (3–4) means the median is 4 and the interquartile range is from 3 to 4. * Statistically significant (*p* < 0.05).

**Table 5:** Quality of medical care in both groups.

		Pandemic Group (*n* = 11)	Non-Pandemic Group (*n* = 11)	*p*
Acute Care LOS, day		16.09 (6.49)	15.36 (4.34)	0.76
PAC LOS, day		32.73 (10.97)	36.64 (12.40)	0.44
Destination after Discharge				1
Household		10 (91%)	10 (91%)	
Nursing Home		1 (9%)	1 (9%)	
Readmission in 14 days	Yes	0 (0%)	0 (0%)	1
6MWD ≥ 205		4 (36%)	3 (27%)	0.66

LOS: length of stay; PAC: post-acute care; 6MWD: 6-min walking distance. Values are expressed as mean (SD) or *n* %.

## Data Availability

The data presented in this study are available on request from the corresponding author.
